# Application of the HRE-S to 140 horses with trigeminal-mediated headshaking and the association of clinical signs with diagnosis, therapy, and outcome

**DOI:** 10.3389/fvets.2024.1329054

**Published:** 2024-04-05

**Authors:** Tanja Kloock, Maren Hellige, Anke Kloock, Karsten Feige, Tobias Niebuhr

**Affiliations:** ^1^Clinic for Horses, University of Veterinary Medicine Hannover, Hanover, Germany; ^2^Department of Cell Biology, NYU Langone Medical Center, New York City, NY, United States

**Keywords:** electric shock-like jerking, history rest and exercise score, equine, pain, welfare

## Abstract

**Background:**

Horses with trigeminal-mediated headshaking (TMHS) exhibit different headshaking patterns (HSPs), electric shock-like jerking, signs of nasal irritation, and painful facial expressions. The History Rest and Exercise Score (HRE-S) was developed to objectively clarify the severity of the condition in affected horses. This score considers the history and severity of clinical signs at rest and exercise. This study aimed to assess the frequency of different clinical signs and their individual associations with diagnosis, treatment, and outcome in horses diagnosed with TMHS.

The clinical records of horses presented with headshaking (HS) at the Clinic for Horses, University of Veterinary Medicine Hannover, between 2006 and 2021 were assessed retrospectively for clinical signs, diagnosis, and treatment. A total of 140 horses were included in the study. Video recordings were evaluated using the HRE-S and compared to the score described by Talbot. Following discharge, owners were interviewed via telephone about the outcome. Correlations between the presence and severity of clinical signs, diagnosis, and outcome were evaluated.

**Results:**

The following clinical signs were significantly correlated with a higher HRE-S and grade by Talbot: HS at walk (independently of HSP) (52.9%, 74/140), increased total number of demonstrated HSP (independent of the dominant HSP) (more than one HSP per horse in 91.4%, 128/140), signs of nasal irritation (75.9%, 104/137), painful facial expression (67.8%, 80/118), and electric shock-like jerking (77.5%, 107/138). Diagnosis and outcome do not correlate with the presence of the above-mentioned clinical signs.

**Conclusion:**

The HRE-S was confirmed as a valid tool to evaluate disease severity in a cohort of 140 horses with HS. Additionally, clinical signs identified as indicators for higher disease severity may have a stronger negative effect on patient welfare, but they do not correlate with diagnosis or outcome.

## 1 Introduction

Trigeminal-mediated headshaking (TMHS) in horses is a condition characterized by sudden and involuntary flicking movements of the head without visible external stimuli (1, 2). In this context, different headshaking patterns (HSPs) are described, with vertical head movements being the most commonly reported sign. Rotatory or horizontal head movements are less commonly reported (2–6). In addition, affected horses often demonstrate electric shock-like jerking, facial signs of pain, and signs of nasal irritation such as increased snorting, nose rubbing, or striking with the forefoot (2–6).

In comparison to healthy horses, the trigeminal nerve of affected horses has a reduced activation threshold and is believed to be functionally impaired (7, 8). In most cases, the reason for trigeminal hypersensitivity remains unidentified. Affected horses are diagnosed with idiopathic TMHS (i-TMHS) (9–11). In contrast, secondary nerve irritation can be caused by a wide variety of different conditions, and such cases are considered to be affected by secondary headshaking (s-HS) (3, 12).

In concordance with trigeminal neuralgia in humans, affected horses are believed to suffer from neuropathic pain and stress, leading to compromised welfare (12, 13). Since clinical signs are believed to be the expression of neuropathic facial pain, welfare impairment is positively linked with the severity of clinical signs. To evaluate the overall disease severity of affected horses, including patient history and clinical signs during rest and exercise, the History Rest and Exercise Score (HRE-S) was established and previously validated as a reliable tool in a cohort of 12 horses (14). It is unknown why the occurrence and frequency of clinical signs vary between different horses and whether clinical signs correlate with outcome and welfare. This study aimed to assess the frequency of different clinical signs and establish their individual associations with welfare, diagnosis, therapy, and outcome in horses diagnosed with TMHS.

## 2 Materials and methods

The digital case records were used to collect the patient history, clinical findings, and results of diagnostic procedures of horses presented with HS referred to the Clinic for Horses at the University of Veterinary Medicine Hannover between 2006 and 2021. The appearance and severity of clinical signs were compared to the diagnosis and outcome.

### 2.1 Horses

Horses were considered for inclusion in the study if they were presented with a history of HS or diagnosed with HS. HS was defined as an intermittent, involuntary, and uncontrolled sudden movement of the head. The diagnosis was based on video recordings obtained during hospitalization. To ensure objective evaluation of clinical signs, video recordings were excluded when one of the following influencing factors was observed: facemasks, nose nets, side reins, or when scientifically proven therapies had been previously administered. These included surgical or medical therapy, neurostimulation, or targeted therapy of findings suspected to be causal (e.g., tooth extraction). Horses were excluded from the study if no video recording met these criteria.

### 2.2 Clinical signs

**Figure 1:**
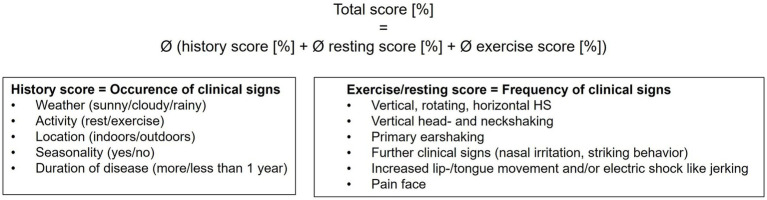
Scoring of horses with HS according to the HRE-S (14). The total score represents the average of the history, resting, and exercise scores. Conclusively, it evaluates the overall disease severity in horses with TMHS, considering the frequency of clinical signs. Every individual score ranges from 0 to 100%, with 0% being no clinical signs and 100% representing the highest possible degree of severity of clinical signs. The resting score for horses with no video records at rest was set at 0%.

Clinical signs of HS were assessed from video recordings and patient records (Table 1). Horses were scored according to the HRE-S (14) to evaluate history (history score), severity of clinical signs during rest and exercise (average resting and average exercise score), as well as overall disease severity (total score) (Figure 1). Therefore, various circumstances were recorded and categorized. These included weather (sunny/cloudy/rainy), activity (exercise/rest), location (outdoors/indoors), seasonality (yes/no), and disease duration (more or less than 1 year since onset). Different HSPs were also assessed according to the HRE-S (14) including vertical, horizontal, or rotating HS, vertical head- and neckshaking, primary earshaking, and HS at walk. Additionally, the total number of demonstrated HSPs and the dominant HSP per horse were documented. In addition, the presence of further clinical signs was evaluated (yes/no): electric shock-like jerking, the presence of a painful facial expression according to the Horse Grimace Scale (HGS) (15), and signs of nasal irritation (increased snorting, nose rubbing, or striking head with forefoot). Usually, horses were not filmed at rest if no clinical signs were present. Therefore, retrospectively, it was presumed that horses without a video recording of a resting period demonstrated no clinical signs at rest. All horses were categorized with the score published by Talbot et al. (16), which evaluates the severity of clinical signs during exercise subjectively and independently of the presence of individual clinical signs: grade 0 (no clinical signs), grade 1 (mild clinical signs), and grades 2 (moderate clinical signs) to 3 (severe clinical signs).

**Table 1:** Clinical signs evaluated from digital patient records for every horse (HS, headshaking, HSP, headshaking pattern).

Conditions	Headshaking	Further clinical signs
Weather (sunny/cloudy/rainy) Activity (exercise/rest) Location (outdoors/indoors) Seasonality (yes/no) Duration (≤1/>1 year)	Headshaking pattern (HSP) Vertical, horizontal, rotating HS Vertical head- and neckshaking Primary earshaking HS at walk (independent of HSP) Total number of demonstrated HSP Dominant HSP	Electric shock-like jerking painful facial expression Signs of nasal irritation

As clinical signs are believed to reflect the expression of neuropathic facial pain, the welfare of affected horses was estimated using the HRE-S, including the evaluation of a painful facial expression according to the HGS by Dalla Costa et al. (15).

### 2.3 Diagnosis

The horses underwent a standardized diagnostic investigation consisting of patient history, blood laboratory analysis (hematology and biochemistry), neurological, ophthalmological, orthopedic, and dental examinations, endoscopy of the upper and lower airways, including guttural pouches, and radiographs of the head and cervical spine. If a pathologic finding within the trigeminal receptive field was identified, local anesthesia of the affected trigeminal branch or targeted therapy of the pathologic lesion was performed (if possible). In cases of a positive outcome (abolished clinical signs), s-HS was diagnosed. If the diagnostic investigation was unremarkable, horses underwent general anesthesia for computed tomography (CT) and/or magnetic resonance imaging (MRI) of the head and otoscopy.

Two groups of horses were diagnosed with i-TMHS: those with no pathological findings in any of the conducted examinations, and those with only mild findings without clinical relevance, commonly observed in healthy horses. Horses were diagnosed with suspected i-TMHS (si-TMHS) when there were mild to moderate findings that could not be evaluated by regional local anesthesia but were assumed not to affect trigeminal nerve function (for example, infundibular caries without pulpitis/alveolitis or cyst-like lesions within the temporomandibular joint). If a finding was verified to be clinically relevant either by a positive response to anesthesia or when clinical signs were abolished following targeted therapy, a diagnosis of s-HS was conducted. For various reasons (such as owner compliance, costs, and technical issues), not all horses in this study underwent all examinations. If questionable findings could neither be clearly diagnosed as significant nor non-significant due to different reasons (such as a missing follow-up, euthanasia, and no local anesthesia/successful therapy performed for a pathology considered relevant), the diagnosis was considered “not evaluable” and horses were excluded from all the analyses regarding association with the diagnosis.

### 2.4 Outcome

Telephone interviews were conducted to obtain information about the outcome. Owners were asked to evaluate the outcome of clinical signs after discharge, including resolution of clinical signs, improvement, unchanged, deterioration, and/or euthanasia related to HS. Additionally, the owners were asked for initial and continued equestrian use (competition, pleasure, retired/breeding, and euthanasia) after discharge. The outcome of equestrian use was then evaluated (improvement, unchanged, deterioration, or euthanasia related to HS). If attempted, responsiveness to scientifically proven therapies [medical (1, 3, 16) or surgical therapy (17, 18), percutaneous electrical nerve stimulation (PENS) (19), or targeted therapy of findings suspected as causal for HS] (20) was assessed (improvement or no improvement).

### 2.5 Statistics

Statistics were performed using R Studio (Version 2022.07.1) as well as GraphPad Prism 9.0 (2020). The association between the HRE-S and the presence of clinical signs, the outcome, and the diagnosis were analyzed using a one-way ANOVA (21) and Tukey’s *post-hoc* test (22), by transforming the data to meet normality requirements. Significances between the presence of clinical signs and diagnosis or outcome were assessed with Pearson’s chi-squared test (23). Correlations between the individual scores of the HRE-S (history, average resting, and average exercise score) were calculated using Spearman’s correlation (24). To assess if the presence of clinical signs had an effect on the score of Talbot et al. (16) Wilcoxon rank sum tests (25) and Kruskal Wallis rank sum tests (26) were performed. Statistical significances are marked: ns: not significant, *p < 0.05, **p < 0.01, ***p < 0.001.

## 3 Results

### 3.1 Horses

A total of 240 horses presented with a history of HS or were diagnosed with HS at the Clinic for Horses, University of Veterinary Medicine Hannover, between 2006 and 2021. Of those patients, 140 horses met the inclusion criteria of this study: diagnosis of HS and existing video recordings without facemasks, nose nets, side reins, or previously administered scientifically proven therapies (see above). Horses were predominantly warmblood breeds, with a median age of 8 years (range 2–21) at presentation. The study population consisted of 97/140 (69.3%) geldings, 41/140 (29.3%) mares, and 2/140 (1.4%) stallions.

### 3.2 Clinical signs

A total of 2,277, with a median of 10 short video recordings per horse (range 1–106), were analyzed during 478 exercise sessions [outdoor arena (*n* = 303), indoor arena (*n* = 171), court (*n* = 4)/ lunging (*n* = 451), free run (*n* = 17), walking (*n* = 10)] and 70 resting periods [stable (n = 41), paddock (n = 27), and pasture (n = 2)].

**Figure 2:**
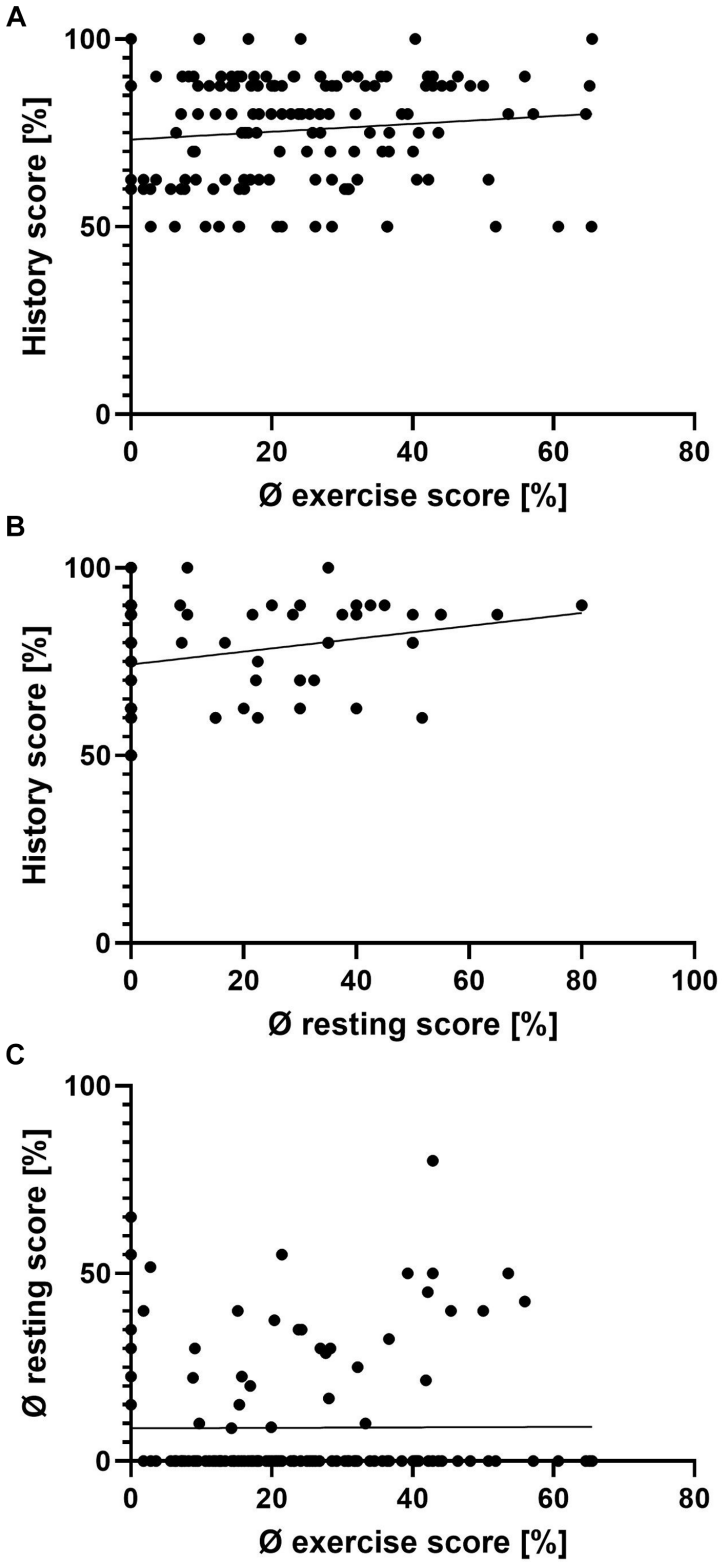
Scatterplots of the history score, average resting score, and average exercise score per individual horse. Average exercise and history score **(A)**, average resting and history score **(B)**, and average exercise and average resting score **(C)**. There is a weak correlation between the history and average resting score **(B)** (Spearman’s rank correlation Rho = 0.18, *p* < 0.05), but none for panel **(A,C)** (Spearman’s rank correlation, *p* > 0.05).

Most horses demonstrated clinical signs only during exercise (92/140, 65.7%). Half of all clinical signs appeared seasonally (50/96, 52.1%). However, the presence of clinical signs was independent of weather (91/127, 71.7%) or location (outdoors/indoors: 88/136, 64.7%) (Table 2). The history score did not correlate with the average resting score or average exercise score per horse, even though statistically there was a weak correlation between the average resting and history score (Spearman’s Rho = 0.18, *p* < 0.05) (Figure 2).

**Table 2:** Frequency [% (*n*)] of conditions under which clinical signs are displayed.

Conditions for demonstrated clinical signs	Frequency [% (*n*)]
Weather	
Permanent Sunny Cloudy Rainy	71,7% (91/127)
	25,2% (32/127)
	1,6% (2/127)
	1,6% (2/127)
Activity	
Exercise Permanent Rest	65,7% (92/140)
	30,7% (43/140)
	3,6% (5/140)
Location	
Permanent Outdoors Indoors	64,7% (88/136)
	30,9% (42)/136
	4,4% (6/136)
Seasonality	
Intermittent	52,1% (50/96)
Permanent	48% (46/96)
Duration	
≤1 year	55,1% (76/138)
>1 year	44,9% (62/138)

Almost all horses included in this study (128/140, 91.4%) exhibited more than one HSP (Table 3). Overall, vertical HS was exhibited by 138/140 (98.6%) of the horses, and it was the predominant type of HSP in 107/136 (78.7%). Independent of HSP, approximately half of the horses (74/140, 52.9%) presented HS during walk. Approximately three-quarters of the horses showed electric shock-like jerking (107/138, 77.5%) and signs of nasal irritation (104/137, 75.9%). Two-thirds presented signs of painful facial expressions (80/118, 67.8%).

**Table 3:** Frequency [% (*n*)] of different clinical signs and their significance for disease severity.

Clinical sign	Significance of correlation to HRE-S	Significance of correlation to score by Talbot et al. (16)	Frequency [% (*n*)]
Demonstrated HSP			
Vertical HS			98.6% (138/140)
Horizontal HS			65.0% (91/140)
Rotating HS			60.7% (85/140)
Primary earshaking			56.4% (79/140)
Vertical head- and neckshaking			46.4% (65/140)
HS at walk	***	***	52.9% (74/140)
Total number of demonstrated HSP	***	***	
4			26.4% (37/140)
2			23.6% (33/1140)
5			20.7% (29/140)
3			20.7% (29/140)
1			8.57% (12/140)
Dominant HSP	ns	ns	
Vertical HS			78,7% (107/136)
>1 HSP			12,5% (17/136)
Primary earshaking			4.4% (6/136)
Horizontal HS			1.5% (2/136)
Rotating HS			1.5% (2/136)
Vertical head- and neckshaking			1.5% (2/136)
Electric shock-like jerking	*	**	77.5% (107/138)
Signs of nasal irritation	***	***	75.9% (104/137)
Painful facial expression	***	**	67.8% (80/118)
Score by Talbot et al. (16)			
Grade 1			32.1% (43/134)
Grade 2			47.0% (63/134)
Grade 3			20.9% (28/134)

Correlations to the total score (HRE-S) and score by Talbot et al. (16) are marked (ns: no significance, *p < 0.05, **p < 0.01, ***p < 0.001). (HS, headshaking, HSP, headshaking pattern).

**Figure 3:**
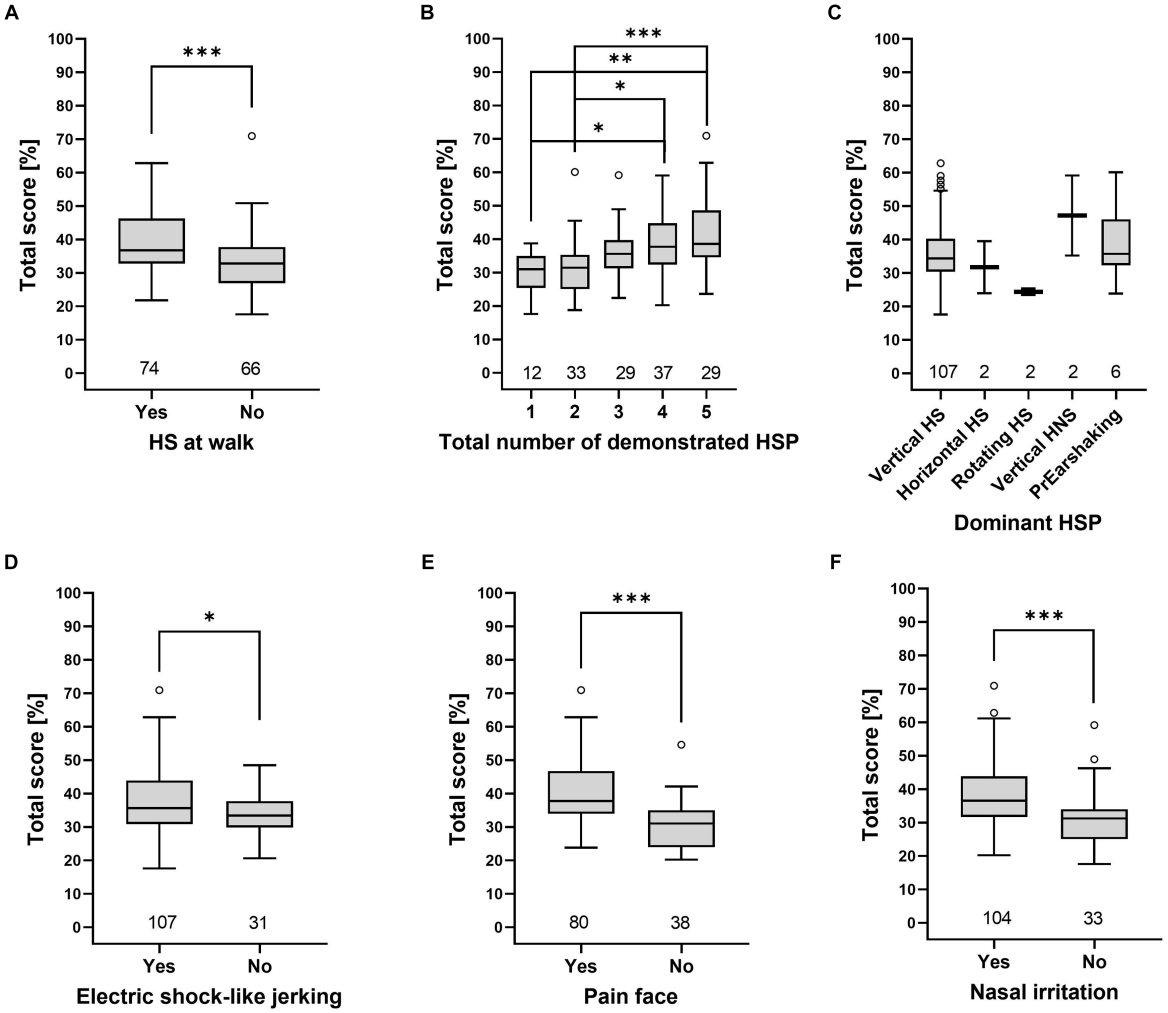
Total score (HRE-S) [%] depends on the presence of individual clinical signs. The total score was higher for the presence of HS at walk **(A)**, a higher total number of demonstrated HSP **(B)**, electric shock-like jerking **(D)**, painful facial expression **(E)**, and nasal irritation **(F)**. In contrast, the total score was independent of the dominant HSP **(C)**. Significance was tested with a one-way ANOVA and Tukey’s *post-hoc* test. Significant differences are marked (*p < 0.05, **p < 0.01, ***p < 0.001) (HNS, head- and neckshaking, HS, headshaking, HSP, headshaking pattern, PrEarshaking, primary earshaking).

**Figure 4:**
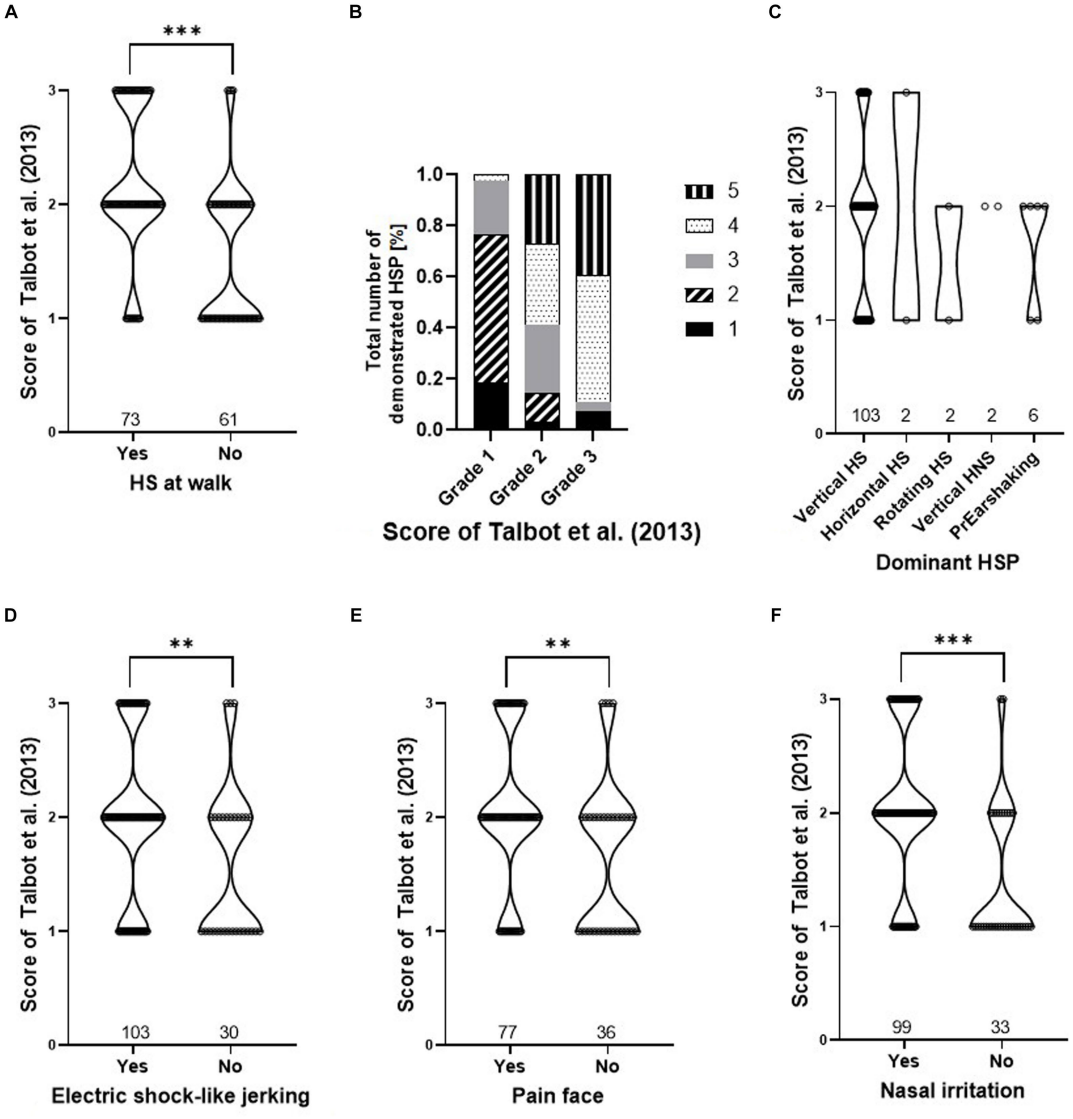
Presence of clinical signs dependent on the score of Talbot et al. (16). Violin plots **(A,C,D–F)** and relative frequencies **(B)** are shown for: HS at walk **(A)**, total number of demonstrated HSP **(B)**, dominant HSP **(C)**, electric shock-like jerking **(D)**, painful face **(E)**, and nasal irritation **(F)**. Effect of the presence of every clinical sign and the score of Talbot et al. (16) for panel **(A,D–F)** were tested with a Wilcoxon rank sum test and are marked as *p < 0.05, **p < 0.01, and ***p < 0.001. The relative frequency of the “total number of demonstrated HSP” **(B)** shows significance to the different grades of Talbot et al. (16) (*p* < 0.001, Pearson’s chi-squared test). There is no significance for the “dominant HSP” **(C)** (*p* > 0.05, Kruskal–Wallis rank sum test). (HNS, head- and neckshaking, HS, headshaking, HSP, headshaking pattern, PrEarshaking, primary earshaking).

Grades by Talbot et al. (16) and total scores of the HRE-S were significantly higher if one of the following clinical signs was present: higher number of HSP, HS at walk, painful facial expressions, signs of nasal irritation, or electric shock-like jerking (*p* < 0.05), while the dominant HSP did not affect either score (Figures 3, 4).

### 3.3 Diagnosis

Overall, most horses (106/112, 94.5%) were considered to be idiopathic, out of which 64/112 (57.1%) were diagnosed with i-TMHS and 42/112 (37.5%) with suspected idiopathic TMHS (si-TMHS). A smaller number of horses (6/112, 5.4%) were diagnosed with s-HS. In 28 horses, a precise diagnosis (i-TMHS, si-TMHS, and s-HS) was “not evaluable.” This was due to the lack of important information such as diagnostic procedures, diagnostic anesthesia, and treatment findings suspected to be causal or missing information about the outcome of treatments. Those horses were excluded from all analyses looking for a correlation between diagnosis and clinical signs or outcomes. There was no correlation between different diagnoses (i-TMHS, si-TMHS, and s-HS) and the HRE-S (*p* > 0.05). In addition, the presence of clinical signs (total number of demonstrated HSP, dominant HSP, HS at walk, painful facial expressions, signs of nasal irritation, and electric shock-like jerking) did not correlate with the diagnosis (*p* > 0.05).

### 3.4 Outcome

The median time span between discharge and telephone interview was 30 months (range 1–176 months). No outcome was obtained in 13/140 (9.2%) of the cases.

Overall, approximately a fifth of the horses showed no more clinical signs after discharge (22/127, 17.3%), approximately a quarter improved (36/127, 28.4%), approximately a third had no change in clinical signs (45/127, 35.5%), and in 24/127 (18.9%), clinical signs deteriorated or led to euthanasia. Equestrian use improved in one horse 1/123 (0.8%), while in just over half of the study population, the equestrian use was unchanged (65/123, 52.8%) or deteriorated (including the ultimate outcome of euthanasia) (57/123, 46.3%). Following scientifically proven therapies, 35/84 (41.7%) of the treated horses improved, whereas 49/84 (58.3%) showed no improvement (Table 4). In 56 cases, no therapy was conducted, or therapeutic success was not evaluable. There was no correlation between the HRE-S and the outcome of clinical signs, the outcome of equestrian use, or the responsiveness to therapy. A detailed outcome of different therapies for the included horses can be found elsewhere (20).

**Table 4:** Frequency [% (*n*)] of outcome.

Outcome	Frequency [% (*n*)]
Response to therapy	
Improvement	41,67% (35/84)
No improvement	58,33% (49/84)
Clinical signs	
Resolution	17,32% (22/127)
Improvement	28,35% (36/127)
Unchanged	35,54% (45/127)
Deterioration/euthanasia	18,90% (24/127)
Equestrian use	
Improvement	0.81% (1/123)
Unchanged	52,84% (65/123)
Deterioration/euthanasia	46,34% (57/1123)

Furthermore, no statistically significant correlation was present between the outcome and the presence of the individual clinical signs: total number of demonstrated HSP, dominant HSP, HS at walk, painful facial expressions, electric shock-like jerking, or nasal irritation.

## 4 Discussion

TMHS in horses is often compared to human trigeminal neuralgia. It is assumed that affected horses suffer from severe neuropathic facial pain (27, 28). Humans with trigeminal neuralgia describe neuropathic pain as a burning, tingling, and electric shock-like sensation that can be triggered by normally pain-free stimuli, such as a light touch to the skin (27). Therefore, clinical signs in horses affected by TMHS are believed to reflect neuropathic facial pain (12, 13). Given these parallels, there is an urgent need for a welfare-oriented assessment method of clinical signs to evaluate horses with TMHS. It is assumed that the severity of clinical signs correlates with the level of pain experienced by the individual. Therefore, the horse’s welfare is less compromised when only rare and very mild clinical signs are apparent. Clinical signs of TMHS can vary widely between affected horses. Some horses are only affected during exercise in spring and fall, whereas other horses show consistently severe clinical signs, even at rest. Welfare is therefore believed to be more compromised in the latter (13). Hence, an emphasis must be placed on analyzing disease severity in greater detail than merely during exercise to evaluate the overall welfare of the diseased horse.

The HRE-S was developed to provide an objective means of grading horses affected by TMHS. The total score provides an objective evaluation of the overall disease severity (14). The presence of clinical signs of TMHS, such as different HSP, signs of nasal irritation, electric shock-like jerking, and an anxious facial expression, are described. However, data focused on the correlation of these signs with disease severity are lacking (2–4, 6, 29–32). A previous study with 12 horses identified signs of nasal irritation, electric shock-like jerking, and painful facial expression as important indicators of a higher severity of TMHS (14). The present study supports those results in a population of 140 horses, as the total score of the HRE-S was significantly higher in horses demonstrating signs of nasal irritation, painful facial expressions, or electric shock-like jerking (Figure 3). In addition, the total score was higher when several HSP were presented by one horse (total number of demonstrated HSP) as well as the appearance of HS at walk (independent of HSP). The appearance of these clinical signs also had a significant effect, leading to higher grades, according to Talbot et al. (16). In contrast to the HRE-S, the grading system of Talbot et al. (16) evaluates disease severity independently of specific clinical signs. Therefore, the following clinical signs can be considered indicators of greater disease severity and compromised welfare in horses with TMHS: HS at walk, increased total number of demonstrated HSP, signs of nasal irritation, painful facial expressions, and electric shock-like jerking.

The existing grading systems for horses with HS assess the intensity of clinical signs during exercise and focus on the impairment of rideability (1, 10, 16). In a previous study, the severity of clinical signs during rest and exercise was not consistent, and neither was the history of horses affected by TMHS (14). The results of the present study are consistent with the previously mentioned study (14) in a greater cohort of 140 horses. The severity of clinical signs during exercise (average exercise score) does not correlate with the severity of clinical signs at rest (resting score) or with the history of the horse (history score). The slight correlation between the resting and history scores is probably due to the fact that the history score is higher when horses demonstrate clinical signs during exercise and rest, as this is one item evaluated in the history score. Therefore, horses should always be monitored with respect to history, rest, and exercise to fully assess the disease severity and the degree of pain. The HRE-S is a valid tool to evaluate disease severity and welfare in horses with TMHS.

Thus far, studies about the impact of the presence and severity of clinical signs on the diagnosis and outcome of horses with TMHS are lacking. In the present study, no correlation was found between the total score of the HRE-S or the presence of clinical signs and diagnosis (i-TMHS, si-TMHS, and s-HS) or outcome. Furthermore, dominant HSP and diagnosis were not correlated. Nevertheless, it needs to be considered that only 12/119 horses demonstrated a dominant HSP other than vertical HS, and only 6/112 horses were diagnosed with secondary HS.

However, it cannot be ruled out that horses diagnosed with i-TMHS without typical clinical signs (vertical HS, nasal irritation, and electric shock-like jerking) are suffering from other types of neuropathies that could not be diagnosed with the standard examination protocol used here. There were only a small number of 6/112 horses diagnosed with s-HS following a detailed and diagnostic investigation in the present study. This suggests that i-TMHS is an independent functional disease and needs to be clearly separated from the small group of horses that have causal primary diseases (s-HS).

The main limitations of this study are the retrospective study design and the caseload of a referral hospital, which included potential bias as horses may have higher degrees of clinical signs in comparison to cases that are successfully managed in the field or via simple owner-based interventions such as the use of nose nets. The retrospective design led to a higher number of cases, and many cases with mild clinical signs were included in this study. Another limitation of the study was the bias in acquiring more videos of severely affected horses. This led to a wide range of short video recordings in the HS group. However, all horses with a history of HS were recorded when exercised. As horses were mainly video recorded when clinical signs were present, it was assumed that horses did not show any signs of HS during rest if there was no video recording in the digital patient record. The resting scores for those horses were set at 0%. Additionally, the follow-up was based on an owner-assessed telephone interview with a wide range of time after discharge that can include owner placebo effects and recall bias. This can be misleading in seasonally affected horses.

## 5 Conclusion

In conclusion, HS at walk, increased total number of demonstrated HSP, signs of nasal irritation, painful facial expression, and electric shock-like jerking were identified as clinical signs of increased disease severity and may have a stronger negative effect on welfare in horses with TMHS. Nevertheless, clinical signs were not associated with diagnosis or outcome. Therefore, no indicators for the outcome or diagnosis of TMHS in horses could be identified. Additionally, the severity of clinical signs during exercise is not correlated with the severity of clinical signs during rest or with the duration of time the horses are affected by the disease. These results highlight the necessity to observe horses at rest and during exercise and take their history into account for an objective evaluation of disease severity and an estimation of animal welfare. The identified indicators can be useful for owners and veterinarians to better assess disease severity and welfare in affected horses.

## Data availability statement

The raw data supporting the conclusions of this article will be made available by the authors, without undue reservation.

## Ethics statement

The animal studies were approved by Prof. Dr. Hiebl, Animal Welfare Officer Member of the Research Ethics Commission of University of Veterinary Medicine Hannover. The studies were conducted in accordance with the local legislation and institutional requirements. Written informed consent was obtained from the owners for the participation of their animals in this study.

## Author contributions

TK: Conceptualization, Data curation, Investigation, Methodology, Writing – original draft, Writing – review & editing, MH: Writing – review & editing, Investigation, Data curation. AK: Investigation, Writing – review & editing, Formal analysis. KF: Supervision, Writing – review & editing, Methodology. TN: Conceptualization, Supervision, Writing – review & editing, Methodology.
